# Modeling dose to normal brain following hypofractionated stereotactic radiotherapy to a single brain metastasis

**DOI:** 10.1002/acm2.70468

**Published:** 2026-02-23

**Authors:** Erin Johns, Catriona Hargrave, Lisa Nissen, Tamara J Barry, Anne Bernard, Mark B Pinkham

**Affiliations:** ^1^ Princess Alexandra Hospital – Ipswich Road, Queensland Health Brisbane Australia; ^2^ Queensland University of Technology Faculty of Health, School of Clinical Sciences Brisbane Australia; ^3^ Princess Alexandra Hospital – Raymond Terrace, Queensland Health Brisbane Australia; ^4^ Institute for Molecular Bioscience University of Queensland Brisbane Australia; ^5^ Faculty of Medicine University of Queensland Brisbane Australia

**Keywords:** brain dose, optimization, Brain metastasis, predictive models, stereotactic radiotherapy

## Abstract

**Background:**

Brain metastases (BM) occur in at least 20% of people with an advanced solid malignancy and can lead to morbidity or mortality if not controlled. Hypofractionated stereotactic radiotherapy (HF‐SRT) may be preferred to single fraction stereotactic radiosurgery (SRS) in certain clinical situations to improve the therapeutic ratio. Certain dose‐volume metrics can predict the risk of symptomatic radionecrosis or other toxicities after HF‐SRT but dosimetric predictors of more complex outcomes such as cognitive function are less well described. For this reason, clinicians aim to limit the dose to uninvolved brain so that it is as low as reasonably achievable rather than simply aiming for a threshold goal. The number of treatment arcs and arc length, floor angles and volumetric modulated arc therapy (VMAT) optimization strategies impact low dose distribution outside the target volume, and various combinations can be trialed and modified during the plan optimization process. On the other hand, variation in patient‐specific factors such as target size and location cannot be changed. This combination of modifiable and fixed factors is expected to influence dose to uninvolved brain, but their relative impacts are not well understood.

**Purpose:**

This study aims to investigate both modifiable and fixed factors influencing low dose distribution to the normal brain and to develop predictive models for multiple normal brain dose metrics used for developing single BM VMAT HF‐SRT plans.

**Methods:**

Consecutive patients receiving HF‐SRT for a single BM or cavity at a single institution between July 2017 and December 2019 were reviewed. Patient‐specific, dosimetric and treatment technique data was collected retrospectively and used to develop statistical models to predict planned dose to the normal brain. Exploratory analyses and relevant parametric and non‐parametric bivariate testing were performed to identify significant variables for inclusion in model development. A manual backward stepwise approach of multiple linear regression (MLR) was used to model achievable low dose in normal brain tissue for three outcomes. The three model outcomes produced in this study include predicting normal brain mean in Gray (Gy), the volume of normal brain receiving 50% of the prescribed dose and the volume of normal brain receiving 25% of the prescribed dose.

**Results:**

Planning data from a total of 90 patients was included in the analysis. Over 60 patient‐specific, dosimetric and treatment technique variables were examined from each patient, with several variables showing significance for all three outcomes through bivariate testing. The nine most significant variables were included in the model development. MLR modeling produced three or four statistically significant variables of planning target volume (PTV) location, PTV shape, PTV volume and arc combinations. Each model explained between 68.6% and 78.3% of the variation in normal brain dose, and the overall significance of each model is *p* < 0.001.

**Conclusions:**

This study has produced three predictive models which estimate an achievable amount of low dose to normal brain for use in the development of single BM VMAT HF‐SRT 6MV plans. This is one of the first studies to investigate the impact of a comprehensive range of variables on achievable low dose distribution.

## 1 INTRODUCTION

Brain metastases (BM) occur in at least 20% of people with an advanced solid malignancy and can lead to morbidity or mortality if not controlled. ^1^, ^2^, ^3^ Stereotactic radiosurgery (SRS) is a standard treatment option for people with a limited number of BM, either as definitive management or in combination with surgery.^1^, ^4^, ^5^ Hypofractionated stereotactic radiotherapy (HF‐SRT) may be preferred to single fraction SRS in certain clinical situations to improve the therapeutic ratio, for example, when treating larger BM, eloquent locations or surgical cavities.^6^, ^7^

Linac‐based HF‐SRT is commonly delivered using volumetric modulated arc therapy (VMAT) which is a highly conformal technique, but exposure of brain tissue beyond the target to radiation is unavoidable.^8^, ^9^ Maximum point doses and dose‐volume metrics can predict the risk of certain complications such as optic neuropathy or radionecrosis after HF‐SRT, but predictors of complex outcomes such as cognitive function are less well described ^10^, ^11^, ^12^ Unlike SRS, where there are established thresholds associated with the volume of healthy brain receiving 10 Gray (Gy), 12 Gy and 14 Gy (V10 Gy, V12 Gy and V14 Gy respectively), reported dose thresholds indicative of toxicity levels after HF‐SRT are varied.^11^ For this reason, clinicians aim to limit the dose to the uninvolved brain to be as low as reasonably achievable, but typically there is no threshold goal. In practice, it can be hard to know when that point has been reached during the dosimetry optimization process.

Human decision‐making is currently inherent to VMAT plan design, and plan quality can vary according to dosimetrist experience.^13^, ^14^ Low dose distribution outside the target volume might vary with arc lengths, arc floor angles or optimization strategies. On the other hand, variation in dose due to patient‐specific factors such as target size and location cannot be changed. The relative contributions and inter‐relations between these combinations of modifiable and fixed factors on low dose to uninvolved brain is not well understood.

Predictive modeling may help facilitate more standardized and efficient workflows, to minimize inter‐user variability, and reduce HF‐SRT planning times to achieve desired dosimetric goals. There is limited literature on predictive modeling in HF‐SRT brain planning. Shiraishi et al.,^15^ published a knowledge‐based predictive model for SRS patients. Bohoudi et al.,^16^ also published a SRS model specific to the volume of normal brain receiving 12 Gy. Ruschin et al.,^17^ investigated the difference in normal brain tissue dose in the HF‐SRT setting when comparing intact lesions and post‐operative brain cavities and their target volumes. A key finding was dose falloff and normal brain dose is largely influenced by planning target volume (PTV) size alone, irrespective of intact or a cavity. However, whether any other factors may influence low dose distribution remains unknown. Ruschin et al.,^18^ expanded on this research to include the impact of number of target lesions, target shape, target volume and comparison between two treatment techniques. They provided a framework for institutions to compare irradiated volume however the goal of the framework was not to provide the most optimal plan but merely to provide technique comparison. These studies considered a limited number of potential predictive factors on low dose. One of the most recent studies published by Tavakoli et al.,^19^ developed regression models for intermediate and low dose wash in intracranial BMs, analyzing total PTV volume and number of metastases. Interestingly, there was no correlation with number of lesions, but PTV volume was a principal predictor of low dose to normal brain. Again, this study only investigates a small number of variables. The aim of the current study was to investigate a wider range of modifiable technical and fixed patient‐specific factors to predict low dose distribution in normal brain to assist dosimetrists with plan generation for HF‐SRT for a single BM.

## 2 MATERIALS AND METHODS

### 2.1 Study cohort and VMAT HF‐SRT technique

Ethical and governance approvals were granted prior to commencement of this single center study (HREC/2022/QMS/76766). Consecutive patients already treated with VMAT HF‐SRT to a single BM or BM cavity between July 2017 and December 2019 were retrospectively identified from a prospectively maintained departmental database. The cohort was limited to single BM, which were predominantly treated with VMAT. Only a small proportion of multiple‐lesion cases were treated with VMAT, as most were managed with the Gamma Knife (Elekta AB, Stockholm, Sweden) system.

HF‐SRT was delivered in the supine position with thermoplastic mask immobilization on an appropriately commissioned linear accelerator (Elekta Versa HD, Elekta, Stockholm, Sweden). Target volume delineation was based on high resolution gadolinium‐enhanced magnetic resonance imaging (MRI) reconstructed into 1.5mm axial slices. This was co‐registered to their 1mm planning computed tomography (CT) scan which extended from the superior aspect of the shell board to inferior of the clavicles. For intact BM, the gross target volume was based on enhancing disease on the T1 weighted MRI sequences and isotropically expanded by 1.5mm to create the PTV. For cavities, the PTV was defined as a 1.5mm expansion on the clinical target volume as described in greater detail previously.^2^

The HF‐SRT dose was 24–30Gy in 3–5 fractions as a covering isodose to the PTV, according to prescribing physician discretion. All plans were created on Pinnacle^3^ (Philips Healthcare, Fitchburg, WI) and calculated on a 2mm dose grid with a 6MV VMAT technique. The collapsed‐cone convolution algorithm was used for heterogeneous dose calculations, and all plans used flattened beams. Dose‐controlling regions of interest (ROIs) including ring structures were used to drive optimization. Typical ROIs generated to optimize the plan included those related to the target volume to meet minimum dose requirements, rings around or at a distance from the edge of target volumes to conform high‐to‐intermediate doses and avoidance ROIs relating to organs at risk (OAR) or normal tissue. Low dose outside the normal tissue tolerance ROI was not routinely controlled. The approach to VMAT plan design and optimization was not standardized during the study period and therefore all plans were constructed according to the discretion of the dosimetrist involved.

### 2.2 Variable selection for model development of normal brain dose

**FIGURE 1:**
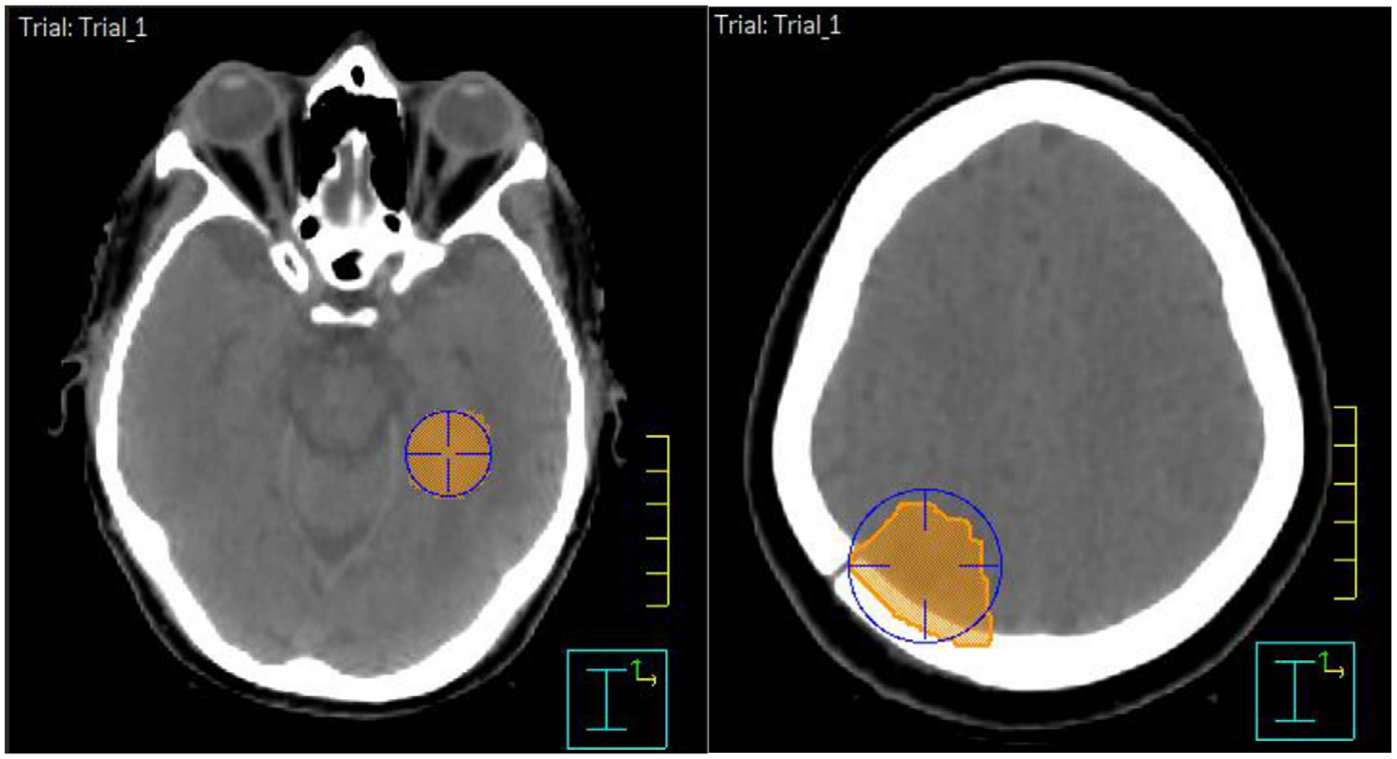
PTV shape; spherical (left) and irregular (right). PTV shape was determined by placing a POI in the epicentre of the PTV and expanded to the closest diameter to visually determine shape.

**FIGURE 2:**
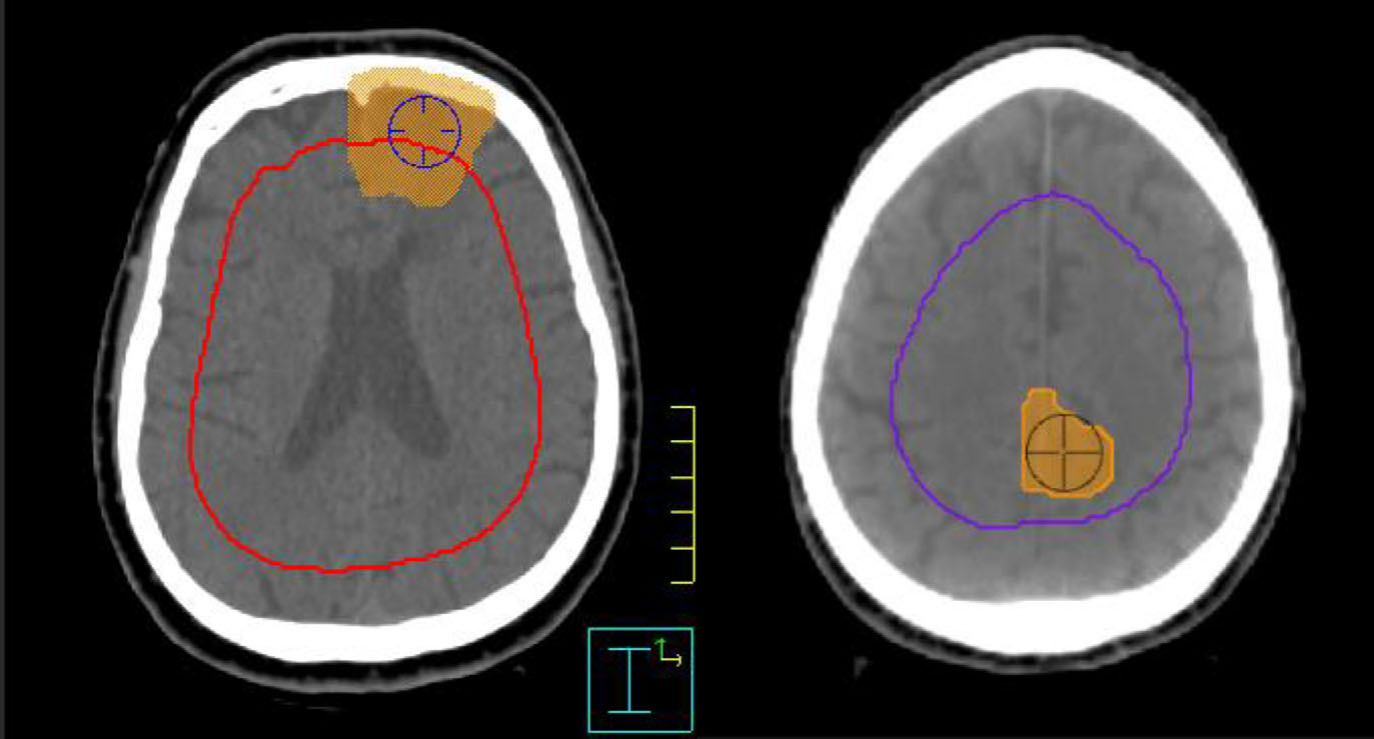
PTV location. Superficial (left) and deep (right). PTV location was determined by creating a Brain‐1.5cm ROI (red contour on the example on the left and and blue contour on the example on the right) and using a POI in the epicentre of the PTV to determine if the point fell within or outside this ROI.

**FIGURE 3:**
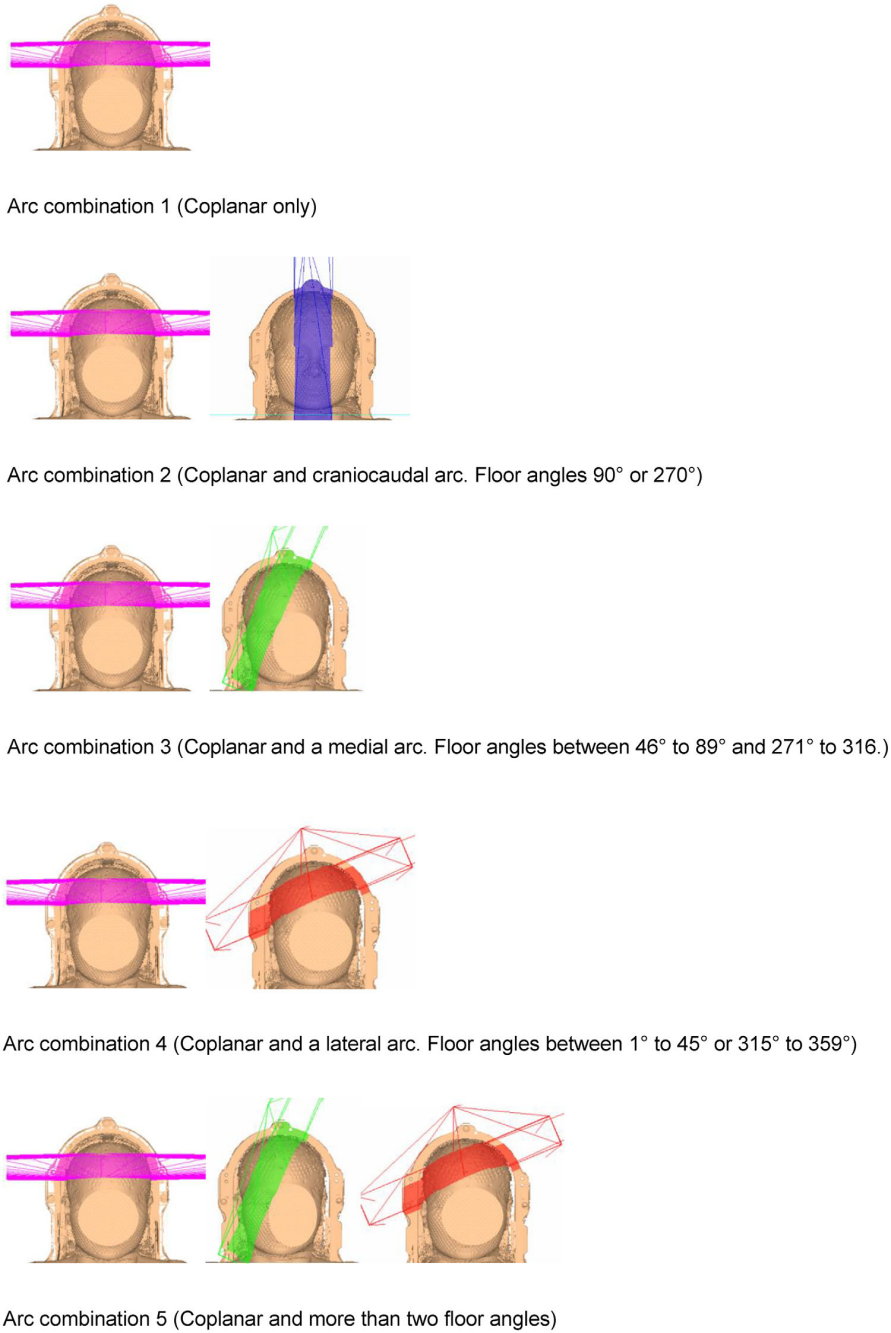
Combinations of different arc sweeps and floor angles. Arc combinations 1–5 from top to bottom.

Normal brain was defined as the entire organ as contoured on planning CT from vertex to foramen magnum, excluding the PTV. For each participant in the cohort, normal brain mean dose and the volume receiving 50% (V50%) and 25% (V25%) of the prescribed dose was recorded. Despite clinically relevant doses of V10 Gy and V12 Gy being reported for SRS^6^, ^10^, ^20^, there are no consistent dose‐volume thresholds reported for HFSRT.^11^, ^21^, ^22^, ^23^ Consequently, the choice of dose‐volume predictors of varying percentages was selected by the authors to allow different fractionation schedules to be compared appropriately and provide a range of low dose distributions. For each participant, over 60 patient‐specific, dosimetric and treatment technique variables were collected. A sample list of these variables can be seen in Table 1. Variable definitions can be found in supplementary materials with the full list of variables (data S1). This large number of variables was investigated to ensure no variables were excluded that may become significant in the modeling methods. A two‐phase statistical analysis was completed. The first stage involved an exploratory analysis, through univariate, bivariate and multivariate testing on IBM SPSS (Version 26). The second stage involved using MLR to model the relationship between the continuous outcomes and explanatory variables determined from the first stage. PTV shape and PTV location were dichotomized into clinically meaningful and simplified groups; spherical versus irregular and superficial versus deep, respectively. Method for categorizing these variables are demonstrated in Figures 1 and 2. Arc combinations considered the treatment couch floor angles and sweep of arc directions and were combined into five clinically meaningful groups for analysis (Figure 3). A competing OAR was defined as any abutting or overlapping with the PTV ROI, including any Planning Organ at Risk Volumes (PRV) which is a small 1.5mm margin added to the OAR to account for organ motion and setup inaccuracies.^24^ No cost functions or plan optimization variables were collected as this often involves multiple iterations with varying workflows and depends on prioritization of clinical risk endpoints. Due to the retrospective nature of this study, assumptions could only be made regarding the iterative clinical decision‐making processes used when determining cost functions and planning system tools.

**TABLE 1:** Sample of variables investigated for model development.

Patient specific variables	Dosimetric variables	Treatment technique variables
Prescription Simultaneous Integrated boost PTV volume PTV location PTV shape Number of OARs? Number of PRVs? Was there a competing OARs/PRVs? Number of competing OARs/PRVs? Normal Brain Volume Brain Volume (vertex to foramen magnum) Brain Avoid Volume (vertex to foramen magnum excluding PTV+1cm)	PTV coverage Plan max Plan max as a percentage Monitor units per fraction Monitor units total Delivery time Brain Avoid max in Gy Brain Avoid max as a percentage Mean normal brain Integral normal brain Mean brain avoid Integral brain avoid Volume of normal brain receiving 50% Volume of normal brain receiving 25% Volume of normal brain receiving 10% Brain Avoid max dose constraint Brain Avoid mean dose constraint	Number of arcs Arc angle start Arc angle finish Collimator angles Number of different collimator angles Floor angles Number of different floor angles Coplanar arc only Arc length Total arc lengths Arc directions Number of ROI rings

Abbreviations: OAR, Organ at risk; PRV, Planning Organ at Risk Volume; PTV, Planning target volume; ROI, Region of Interest.

### 2.3 Statistical analyses

Descriptive statistics and bivariate tests were completed on IBM SPSS Statistics (Version 26). Normality of continuous data was assessed using the Shapiro‐Wilk test. Parametric and non‐parametric tests were used to assess for correlations between the collected fixed and modifiable variables and the three outcomes of normal brain mean dose, V50% and V25% of the prescribed dose (data S2).

Specific statistical tests were selected based on variable type and distribution to ensure appropriate analysis. Pearson's correlation was used for normally distributed continuous variables, such as PTV volume and total arc length, and Spearman's correlation for non‐parametric continuous data. Independent *t*‐tests were applied to compare parametric two‐group categorical variables, while Mann–Whitney *U* tests were used for non‐parametric two‐group comparisons, including variables such as tumor shape and the presence of a competing OAR. Comparisons involving three or more groups, such as different arc‐combination or floor‐angle categories, were performed using ANOVA for parametric data or the Kruskal–Wallis test for non‐parametric data. Only the final statistical outputs are presented, as intermediate screening steps did not alter variable inclusion. Variables with *p* < 0.15 were selected to be included in the MLR. This *p* value was selected to ensure an adequate number of variables were investigated.

### 2.4 Model development

The General Linear Model function in SPSS was used to create the MLR models. A manual backward stepwise approach was employed to select variables in the final model. The nine most significant variables with the outcome of normal brain mean in Gy were included in the model development as this is a primary planning goal. These nine variables were selected out of the 60 based on the exploratory analysis and the parametric and non‐parametric bivariate testing results of *p* < 0.15. Initially, all nine variables were included and then eliminated one‐by‐one after each model iteration. A variable was eliminated when it had the largest p value that was not significant (*p* > 0.05). The adjusted R^2^ was also analyzed after each model iteration (see data S3). The adjusted *R* ^2^ represents the variation in the observed outcome that is explained by the model, ranging from 0 to 1. Generally, a higher value indicates a better fit of the model however this is a caveat as the adjusted *R* ^2^ will increase with an increasing number of explanatory variables. Therefore, it is important the parsimony principle is employed, ensuring a balance of the adjusted *R* ^2^ and explanatory variables so the best fitted model is selected.^25^

The Akaike Information Criterion (AIC) is another statistical value to assist in determining how well the model fits and was generated in the R statistical software (https://www.r‐project.org/) using the *lm* and *AIC* function. The AIC is insignificant on its own but can be used to compare models with the same outcomes.^26^ The lower the AIC means the fit of the model is improving which was the method employed to evaluate model performance. The standardized coefficients for each model were also generated using the *lm.beta* function to identify which variables have the most influence.

All model assumptions regarding independent variables, linearity, equal variances, normally distributed residuals, and multi‐collinearity were checked.

## 3 RESULTS

### 3.1 Study cohort

There were 90 patients with a single BM or cavity receiving VMAT HF‐SRT included in the modeling. Mean brain volume was 1432.5 cm^3^ (range 1077.4‐1875.8, SD ± 163). Mean PTV volume was 30.3 cm^3^ (range 1.4‐174, SD ± 26.8). The prescribed doses were 24 Gy in 3 fractions (21%), 25 Gy in 5 fractions (21%), 27.5 Gy in 5 fractions 47%) and 30 Gy in 5 fractions (11%). The average normal brain mean dose was 2.5 Gy (range 0.6‐5.2, SD ± 1.0). The average normal brain V50% was 40.7 cm^3^ (range 5.9‐111.6, ± SD 21.4) and V25% was 127.5 cm^3^ (Range 16–365.6, SD ± 67.7), respectively. Additional details pertaining to technical variables for HF‐SRT in the cohort are shown in Table 2.

**TABLE 2:** Baseline characteristics.

Variable		*N*	%
*PTV location*	Deep	34	38%
	Superficial	56	62%
*PTV shape*	Spherical	19	21%
	Irregular	71	79%
*Arc combination*	Arc combination 1	9	10%
	Arc combination 2	42	47%
	Arc combination 3	14	16%
	Arc combination 4	19	21%
	Arc combination 5	6	6%
*Was there a competing OAR*	Yes	16	18%
	No	74	82%
*Total number of different floor angles*	1	9	10%
	2	75	83%
	3	6	7%
*Total number of different collimator angles*	1	45	50%
	2 or 3	45	50%
*Total number of arcs*	1 or 2	60	67%
	3 or 4	30	33%

*Note*: Arc combinations 1–5 defined in Figure 3.

Abbreviations: OAR, Organ at risk; PTV, Planning target volume.

### 3.2 Variable selection for model development

The nine most significant variables from the bivariate testing (*p* < 0.15) with the outcome of normal brain mean in Gy included in MLR model development are shown in Table 3. Six of the variables were also significant for V50% and V25% with some interchange for the remaining three variables.

**TABLE 3:** Significant variables from bivariate testing for normal brain mean outcome.

Variable	Fixed or Modifiable	Statistical test	*p* value
PTV volume	Fixed	Pearson correlation	*R* = 0.78, *p* < 0.001
PTV shape	Fixed	Mann–Whitney	*p* < 0.001
PTV location	Fixed	Independent *t*‐test	*p* < 0.001
Competing OAR	Fixed	Mann–Whitney	*p* = 0.046
Arc combinations	Modifiable	ANOVA	*p* = 0.127
Floor angles	Modifiable	Kruskal–Wallis	*p* = 0.07
Total arc length	Modifiable	Pearson correlation	*R* = 0.39, *p* < 0.001
Number of arcs	Modifiable	Independent *t*‐test	*p* = 0.001
Collimator angles	Modifiable	Independent *t*‐test	*p* = 0.08

Abbreviations: OAR, Organ at risk; PTV, Planning target volume.

### 3.3 The fitted models

The final models included three or four variables: PTV volume, PTV location, PTV shape and arc combinations. The *p*‐values of these variables from the MLR modeling can be seen in Table 4 for all three models.

**TABLE 4:** MLR statistically significant variables.

Normal Brain Mean	Fixed or Modifiable	*p*‐value
PTV volume	Fixed	*p* < 0.004
PTV shape	Fixed	*p* < 0.000
PTV location	Fixed	*p* < 0.001
**V50%**	**Fixed or Modifiable**	***p*‐value**
PTV volume	Fixed	*p* < 0.001
PTV shape	Fixed	*p* = 0.001
PTV location	Fixed	*p* < 0.001
Arc Combinations	Modifiable	*p* = 0.003
**V25%**	**Fixed or Modifiable**	***p*‐value**
PTV volume	Fixed	*p* < 0.001
PTV shape	Fixed	*p* = 0.019
PTV location	Fixed	*p* < 0.001
Arc combinations	Modifiable	*p* = 0.035

Abbreviation: PTV, Planning target volume.

The fitted models are presented below. Coding outputs relevant to the models are shown in Table 5.

**TABLE 5:** Coding for models.

Variable	Coding for models
PTV volume	Volume of PTV in cm^3^
PTV location	0 = Superficial [referent] 1 = Deep
PTV shape	0 = Irregular [referent] 1 = Spherical
Arc combinations	1 = AC1 (Arc combination 1) 1 = AC2 (Arc combination 2) 1 = AC3 (Arc combination 3) 1 = AC4 (Arc combination 4) 0 = AC5 (Arc combination 5) [referent] *remove any unused variable from equation

Abbreviation: PTV, Planning target volume.

#### 3.3.1 Normal brain mean

(1) $$\begin{array}{c} \text{Normal Brain Mean in Gy}=1.70\;+\;0.55*\text{Location}\;\\ -\;0.47*\text{Shape}\;+\;0.02*\text{Volume} \end{array}$$

#### 3.3.2 Volume of normal brain receiving 50% of the prescribed dose

(2) $$\begin{array}{c} V50\%\;\text{of prescribed dose}=17.86+10.48*\text{Location}\\ -\;9.70*\text{Shape}\;+\;7.45*\text{AC}1\;+\;0.20*\text{AC}2\;\\ +\;11.94*\text{AC}3\;-\;0.82*\text{AC}4\;+\;0.61*\text{Volume} \end{array}$$

#### 3.3.3 Volume of normal brain receiving 25% of the prescribed dose

(3) $$\begin{array}{c} V25\%\;\text{of}\;\text{prescribed}\;\text{dose}=53.50+39.61^{*}\text{Location}\\ \text{--}24.71^{*}\text{Shape}+30.31^{*}\text{AC}1+3.02^{*}\text{AC}2+28.06^{*}\text{AC}3\\ \text{--}5.25^{*}\text{AC}4+1.87^{*}\text{Volume} \end{array}$$

### 3.4 Model evaluation

All three of the models were significant (*p* < 0.001). The Adjusted R^2^ for the normal brain in mean model (Equation 1) was 0.686, meaning 68.6% of the variation in normal brain mean is explained by the model. This model had an AIC value of −105.8. The Adjusted R^2^ for the V50% model (Equation 2) was 0.783, meaning 78.3% of the variation in volume of normal brain receiving 50% of the prescribed dose is explained by the model. This model had an AIC value of 421.4. Lastly, the Adjusted *R^2^* for the V25% model (Equation 3) was 0.731. So, 73.1% of the variation in volume of normal brain receiving 25% of the prescribed dose is explained by the model. This model had an AIC value of 648.9. All MLR model assumptions for each model were met. The variables and their standardized coefficients from most influential to least influential for the three models can be seen in Table 6. PTV volume and superficial location of PTV are the two most influential variables in all three models.

**TABLE 6:** Standardized Coefficients.

	Order of variable and standardized coefficients						
Normal brain mean	PTV volume 0.65	Superficial PTV 0.28	Irregular PTV −0.19	N/A	N/A	N/A	N/A
V50%	PTV volume 0.76	Superficial PTV 0.24	Arc combination 3 0.20	Irregular PTV −0.19	Arc combination 1 0.1	Arc combination 4 −0.02	Arc combination 2 0.01
V25%	PTV volume 0.74	Superficial PTV 0.28	Irregular PTV −0.15	Arc combination 3 0.15	Arc combination 1 0.13	Arc combination 4 0.03	Arc combination 2 0.03

*Note*: Arc combinations 1–5 defined in Figure 3.

Abbreviation: N/A, Not Applicable; PTV, Planning target volume

**FIGURE 4:**
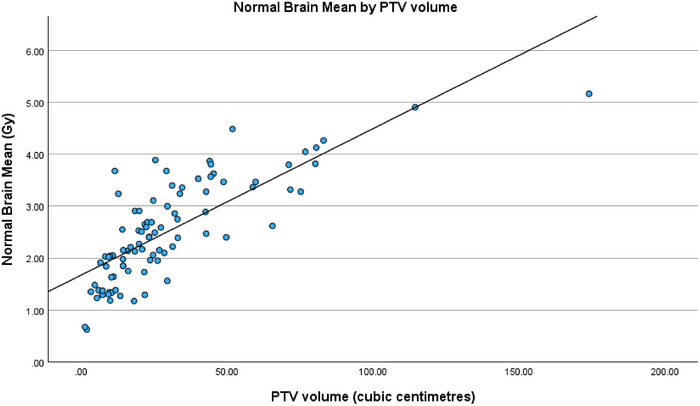
Regression fit of normal brain mean (Gy) by PTV volume (cm^3^).

**FIGURE 5:**
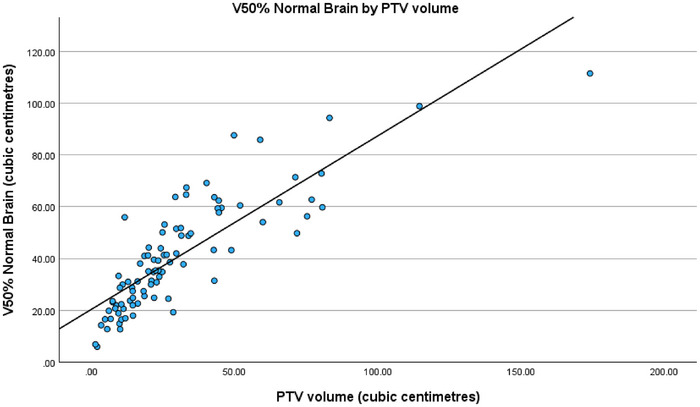
Regression fit of V50% normal brain (cm^3^) by PTV volume (cm^3^).

**FIGURE 6:**
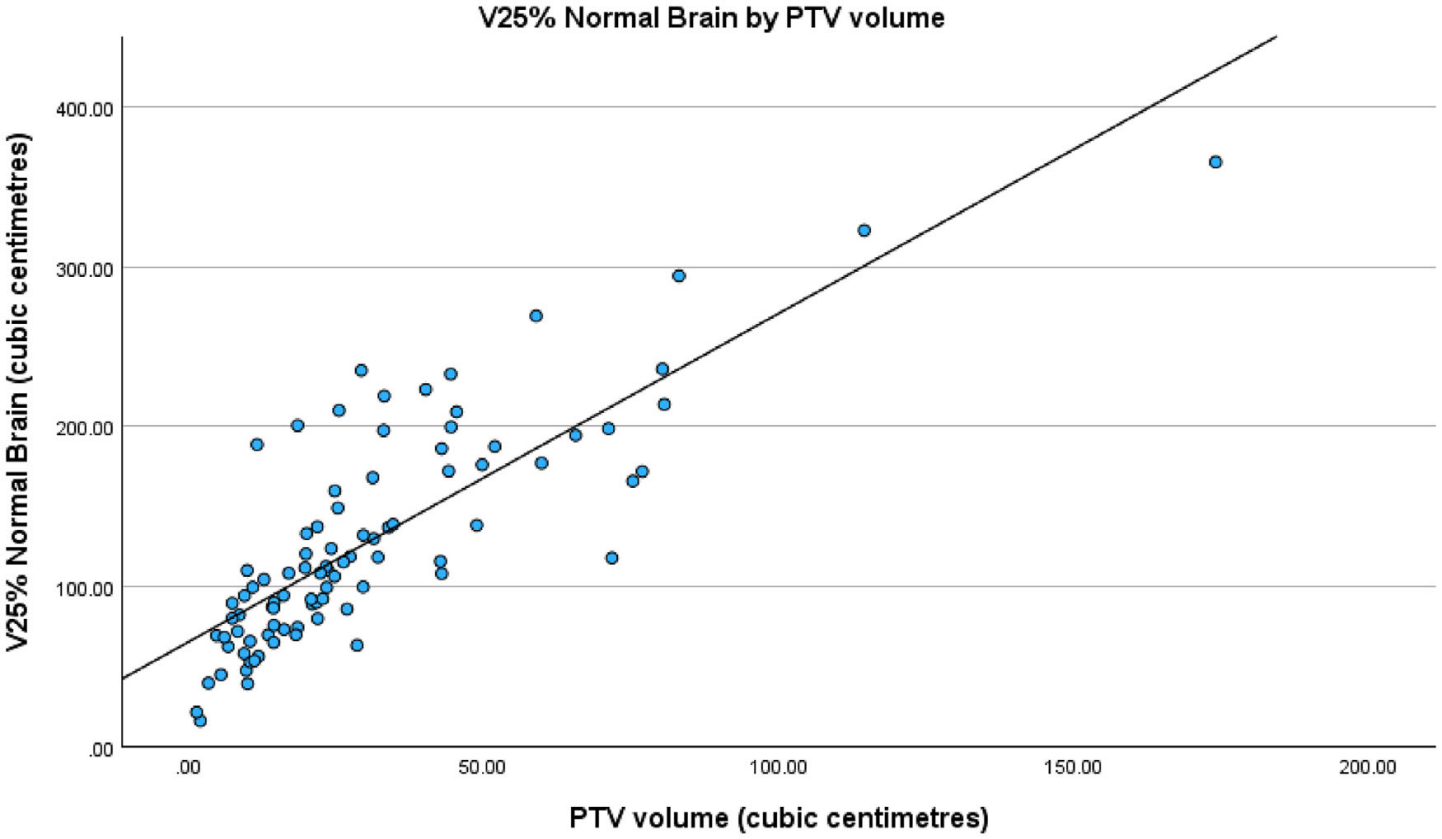
Regression fit of V25% normal brain (cm^3^) by PTV volume (cm^3^).

When analyzing the regression fits of the three models with PTV volume, a strong positive correlation can be seen as displayed in Figures 4, 5 and 6. Unsurprisingly, increases in PTV volume were associated with higher mean normal brain dose as well as larger V50% and V25% normal brain volumes.

## 4 DISCUSSION

This study aimed to investigate both fixed and modifiable variables on low dose distribution to normal brain for patients receiving VMAT HF‐SRT, and then create predictive models for three dose metrics of mean dose, V50% and V25%. The models presented explain a good range of the observed variation in the outcome, between 68.6% to 78.3% with all models being significant (*p* < 0.001). The remaining unexplained variation may reflect the influence of outliers, planning cost functions not captured in this study, and other variables such as proximity of the PTV to competing OARs. While these latter factors did not demonstrate significance within the current dataset, they may become more apparent in larger cohorts and warrant further investigation.

As there is limited literature on using MLR for predictive modeling in radiation therapy, it is difficult to provide context on how well these models perform. However, one study by Gui et al.,^27^, ^28^ included two models which predict changes in measures of verbal memory and changes in whole brain volume at 6 months after whole brain radiation therapy. The model predicting change in verbal memory produced an adjusted R^2^ value of 0.35 and the other model predicting change in whole brain volume produced an *R* ^2^ value of 0.34. Whilst the study only chose to use two explanatory variables, these adjusted *R* ^2^ values are much lower than the ones reported in this research of 0.68, 0.72, and 0.78. It is anticipated that the models will identify a dose which the radiation therapist (RT) planners and radiation oncologist's (ROs) can use as a guide to what may be as low as reasonably achievable. For the RT, this saves time in the planning phase knowing when to stop optimizing the plan and for the ROs, the model outcomes can give an indication that low doses to the normal brain might not be improved further. A follow up study validating the models and the clinical implementation framework is currently being finalized. The anticipated clinical benefits of using the models may potentially reduce negative neurocognitive effects caused by radiation to healthy brain tissue.

The models contain three or four fixed and modifiable variables that predict low dose to normal brain; PTV volume, PTV shape, PTV location, and arc combinations. When analyzing the standardized coefficients for each of the variables in the model, PTV volume is by far the most influential variable in all three models (Table 6). For normal brain mean dose, V50% and V25%, the standardized coefficients were 0.65, 0.76, and 0.74, respectively. This suggests PTV volume has the largest impact on low dose which is consistent with the recent findings of Tavakoli et al.^19^ where PTV volume emerged as the strongest predictor of low dose. This finding highlights the importance of reducing PTV margins where possible, considering dose to normal brain within and beyond the target volume.

**FIGURE 7:**
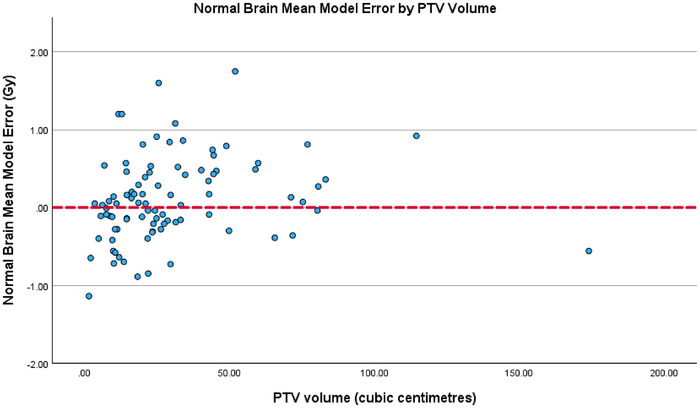
Normal brain mean model error (Gy) by PTV volume (cm^3^).

Although PTV volume emerged as the most influential predictor based on its standardized coefficient, the residual analysis displayed in Figure 7 indicates that it does not introduce systematic bias. The residuals plotted against PTV volume show no systematic trend, with errors distributed evenly across the full volume range. While greater variability is noted at larger PTV's, the absence of directional deviation from the zero‐error line supports the assumption of homoscedasticity. Thereby, reinforcing the model's robustness across varying target sizes.

The choice of mean brain dose, V50%, and V25% to reflect low dose in the brain may need to be revisited in the future as more evidence on the relationship between these metrics and patient outcomes become available. However, they were selected for this study as mean brain dose is a primary planning goal in HF‐SRT and the V50% and V25% are indicative of lower doses which allow different dose fractionations to be compared appropriately. Ruschin et al.,^17^ recorded mean dose to normal brain for intact lesions receiving 30 Gy in five fractions of 2.9 Gy (ranging from 1.0–8.4) and for cavities 3.4 Gy (ranging from 1.5–6.5). The mean dose to normal brain for intact lesions receiving 27.5 Gy in 5 fractions was 3.8 Gy (ranging from 1.1‐8.6) and for cavities was 5 Gy (ranging from 2.0–7.1). Lastly, for intact lesions for 25 Gy in 5 fractions the mean dose was 3.2 Gy (ranging from 1.1–7.2) and for cavities 3.9 Gy (ranging from 1.0‐6.5). Ruschin et al.,^17^ defined normal brain as the volume of the whole brain tissue minus all CTVs. The study also included multiple BM which resulted in larger mean doses. This makes it challenging to compare findings with this research. In a subsequent paper, Ruschin et al.,^18^ provided a framework for differing volumes of normal brain receiving percentages of the prescribed dose down to 50%. They included V50% as this represents a clinically relevant lower limit to compare dose falloff which consequently was a consideration in selecting V50% as one of the modeling outcomes in the current study.^18^, ^29^ 50% dose clouds have also been used in another study as an intermediate dosimetric quantity.^19^

A strength of the current study is the range of variability within the model training cohort, thus improving generalizability. For example, the PTV volumes in this study range from 1.4 cm^3^ to 174 cm^3^ with the mean volume being 30.3 cm^3^ (SD ± 26.8). This suggests the model would be most accurate for the cases where the PTV volume ranges from 3.5 cm^3^ to 57.0 cm^3^ and may potentially over‐ or underestimate outside this. Nonetheless, this provides reassurance the models will be mostly accurate for this range of PTV volumes.

As mentioned above, a possible limitation is the choice of modelled outcomes because there is limited information on which subunits or dose‐volume combinations are best to measure for HF‐SRT from the literature. A further challenge is the categorization of some of the modifiable variables which may be oversimplified and therefore not provide a true representation of their influence on low dose distribution in the brain. Additionally, the low numbers in some categories will reduce power to identify a true statistically significant correlation. Validation on an external data to confirm model accuracy in a different treatment planning system with different beam models and new clinicians prescribing potentially different doses would be helpful.

## 5 CONCLUSION

This study has produced three predictive models which estimate an achievable amount of low dose to normal brain tissue for single BM VMAT HF‐SRT 6MV plans. The models include three or four statistically significant variables of: PTV volume, PTV location, PTV shape and arc combinations. This is one of the first studies to investigate both fixed and modifiable variables on low dose distribution.

## AUTHOR CONTRIBUTION

Erin Johns conceived and designed the research and wrote the manuscript. Anne Bernard assisted Erin Johns with statistical analysis. Mark B Pinkham, Tamara J Barry, Lisa Nissen, and Catriona Hargrave provided critical feedback on the manuscript. All authors reviewed and approved the final version.

## ETHICAL STATEMENT

Ethical and governance approvals were granted prior to commencement of this single center study (HREC/2022/QMS/76766).

## CONFLICT OF INTEREST STATEMENT

The authors have no relevant conflicts of interest to disclose.

## Supporting information



Supporting Information

## References

[1] Andrews DW , Scott CB , Sperduto PW , et al. Whole brain radiation therapy with or without stereotactic radiosurgery boost for patients with one to three brain metastases: phase III results of the RTOG 9508 randomised trial. Lancet. 2004;363(9422):1665‐1672. doi:10.1016/S0140‐6736(04)16250‐8 15158627 10.1016/S0140-6736(04)16250-8

[2] Garimall S , Shanker M , Johns E , et al. Evidence of dose‐response following hypofractionated stereotactic radiotherapy to the cavity after surgery for brain metastases. J Neuro‐oncol. 2020;146(2):357‐362. doi:10.1007/s11060‐019‐03383‐w 10.1007/s11060-019-03383-w 31907796

[3] Loo M , Clavier J‐B , Khalifa JA , Moyal E , Khalifa J . Dose‐response effect and dose‐toxicity on stereotactic radiotherapy for brain metastases: a review. Cancers. 2021;13(23):6086. doi:10.3390/cancers13236086 34885193 10.3390/cancers13236086 PMC8657210

[4] Kocher M , Soffietti R , Abacioglu U , et al. Adjuvant whole‐brain radiotherapy versus observation after radiosurgery or surgical resection of one to three cerebral metastases: results of the EORTC 22952–26001 study. J Clin Oncol. 2011;29(2):134‐41. doi:10.1200/JCO.2010.30.1655 21041710 10.1200/JCO.2010.30.1655 PMC3058272

[5] Mahajan A , Ahmed S , McAleer MF , et al. Post‐operative stereotactic radiosurgery versus observation for completely resected brain metastases: a single‐centre, randomised, controlled, phase 3 trial. Lancet Oncol. 2017;18(8):1040‐1048. doi:10.1016/S1470‐2045(17)30414‐X 28687375 10.1016/S1470-2045(17)30414-X PMC5560102

[6] Minniti G , Scaringi C , Paolini S , et al. Single‐fraction versus multifraction (3 × 9 Gy) stereotactic radiosurgery for large (>2 cm) brain metastases: a comparative analysis of local control and risk of radiation‐induced brain necrosis. Int J Radiat Oncol Biol Phys. 2016;95(4):1142‐1148. doi:10.1016/j.ijrobp.2016.03.013 27209508 10.1016/j.ijrobp.2016.03.013

[7] Pinkham MB , Whitfield GA , Brada M . New developments in intracranial stereotactic radiotherapy for metastases. Clin Oncol (R Coll Radiol). 2015;27(5):316‐23. doi:10.1016/j.clon.2015.01.007 25662094 10.1016/j.clon.2015.01.007

[8] Brun L , Dupic G , Chassin V , et al. Hypofractionated stereotactic radiotherapy for large brain metastases: optimizing the dosimetric parameters. Cancer Radiother. 2021;25(1):1‐7. doi:10.1016/j.canrad.2020.04.011 33257109 10.1016/j.canrad.2020.04.011

[9] Andrevska A , Knight KA , Sale CA . The feasibility and benefits of using volumetric arc therapy in patients with brain metastases: a systematic review. J Med Radiat Sci. 2014;61(4):267‐276. doi:10.1002/jmrs.69 25598981 10.1002/jmrs.69 PMC4282127

[10] Minniti G , Clarke E , Lanzetta G , et al. Stereotactic radiosurgery for brain metastases: analysis of outcome and risk of brain radionecrosis. Radiat oncol (London, England). 2011;6(1):48‐48. doi:10.1186/1748‐717X‐6‐48 10.1186/1748-717X-6-48 PMC3108308 21575163

[11] Milano MT , Grimm J , Niemierko A , et al. Single‐ and multifraction stereotactic radiosurgery dose/volume tolerances of the brain. Int J Radiat Oncol Biol Phys. 2021;110(1):68‐86. doi:10.1016/j.ijrobp.2020.08.013 32921513 10.1016/j.ijrobp.2020.08.013 PMC9387178

[12] Pinkham MB , Sanghera P , Wall GK , Dawson BD , Whitfield GA . Neurocognitive effects following cranial irradiation for brain metastases. Clin Oncol (R Coll Radiol). 2015;27(11):630‐639. doi:10.1016/j.clon.2015.06.005 26119727 10.1016/j.clon.2015.06.005

[13] Berry SL , Boczkowski A , Ma R , Mechalakos J , Hunt M . Interobserver variability in radiation therapy plan output: results of a single‐institution study. Pract Radiat Oncol. 2016;6(6):442‐449. doi:10.1016/j.prro.2016.04.005 27374191 10.1016/j.prro.2016.04.005 PMC5099085

[14] Nelms BE , Robinson G , Markham J , et al. Variation in external beam treatment plan quality: an inter‐institutional study of planners and planning systems. Pract Radiat Oncol. 2012;2(4):296‐305. doi:10.1016/j.prro.2011.11.012 24674168 10.1016/j.prro.2011.11.012

[15] Shiraishi S , Tan J , Olsen LA , Moore KL . Knowledge‐based prediction of plan quality metrics in intracranial stereotactic radiosurgery: knowledge‐based prediction of plan quality metrics in SRS. Med Phys (Lancaster). 2015;42(2):908‐917. doi:10.1118/1.4906183 10.1118/1.4906183 25652503

[16] Bohoudi O , Bruynzeel AM , Lagerwaard FJ , Cuijpers JP , Slotman BJ , Palacios MA . Isotoxic radiosurgery planning for brain metastases. Radiother Oncol. 2016;120(2):253‐7. doi:10.1016/j.radonc.2016.05.001 27212141 10.1016/j.radonc.2016.05.001

[17] Ruschin M , Lee Y , Beachey D , et al. Investigation of dose falloff for intact brain metastases and surgical cavities using hypofractionated volumetric modulated arc radiotherapy. Technol Cancer Res Treat. 2016;15(1):130‐8. doi:10.1177/1533034614567277 25627201 10.1177/1533034614567277

[18] Ruschin M , Sahgal A , Soliman H , et al. Investigation of irradiated volume in linac‐based brain hypo‐fractionated stereotactic radiotherapy. Radiat Oncol. 2017;12(1):117. doi:10.1186/s13014‐017‐0853‐5 28709427 10.1186/s13014-017-0853-5 PMC5513379

[19] Tavakoli M , Wadi‐Ramahi S , Ashmeg S , Lalonde R , Siddiqui Z . Tumor‐driven SRS VMAT planning: regression models for intermediate and low dose spillage. J Appl Clin Med Phys. 2025;26(8):e70184. doi:10.1002/acm2.70184 40744900 10.1002/acm2.70184 PMC12313394

[20] Blonigen BJ , Steinmetz RD , Levin L , Lamba MA , Warnick RE , Breneman JC . Irradiated volume as a predictor of brain radionecrosis after linear accelerator stereotactic radiosurgery. Int J Radiat Oncol Biol Phys. 2010;77(4):996‐1001. doi:10.1016/j.ijrobp.2009.06.006 19783374 10.1016/j.ijrobp.2009.06.006

[21] Minniti G , Esposito V , Clarke E , et al. Multidose stereotactic radiosurgery (9 Gy × 3) of the postoperative resection cavity for treatment of large brain metastases. Int J Radiat Oncol Biol Phys. 2013;86(4):623‐9. doi:10.1016/j.ijrobp.2013.03.037 23683828 10.1016/j.ijrobp.2013.03.037

[22] Minniti G , D'Angelillo RM , Scaringi C . et al. Fractionated stereotactic radiosurgery for patients with brain metastases. J Neurooncol. 2014;117(2):295‐301. doi:10.1007/s11060‐014‐1388‐3 24488446 10.1007/s11060-014-1388-3

[23] Ernst‐Stecken A , Ganslandt O , Lambrecht U , Sauer R , Grabenbauer G . Phase II trial of hypofractionated stereotactic radiotherapy for brain metastases: results and toxicity. Radiotherapy and oncology. 2006;81(1):18‐24. doi:10.1016/j.radonc.2006.08.024 16978720 10.1016/j.radonc.2006.08.024

[24] International Commission on Radiation Units and Measurements . Prescribing, recording and reporting photon beam therapy (supplement to ICRU Report 50). Journal of the ICRU. 1999;1(3):ICRU Report 62.

[25] Kuppermann N , Willits N , In response to “Statistical Models and Occam's Razor”. Acad Emerg Med. 2000;7(1):100‐3. doi:10.1111/j.1553‐2712.2000.tb01905.x 10.1111/j.1553-2712.2000.tb01905.x 10894254

[26] Gevaert T , Steenbeke F , Pellegri L , et al. Evaluation of a dedicated brain metastases treatment planning optimization for radiosurgery: a new treatment paradigm? Radiat oncol (London, England). 2016;11(1):13‐13. doi:10.1186/s13014‐016‐0593‐y 10.1186/s13014-016-0593-y PMC4736109 26831367

[27] Gui C , Chintalapati N , Hales RK , et al. Reduction in whole brain volume is associated with decline in verbal memory following prophylactic cranial irradiation for limited‐stage small‐cell lung cancer. Int J Radiat Oncol Biol Phys. 2019;105(1):S11‐S12. doi:10.1016/j.ijrobp.2019.06.401

[28] Gui C , Grimm J , Kleinberg LR , et al. A dose‐response model of local tumor control probability after stereotactic radiosurgery for brain metastases resection cavities. Adv Radiat Oncol. 2020;5(5):840‐849. doi:10.1016/j.adro.2020.06.007 33083646 10.1016/j.adro.2020.06.007 PMC7557194

[29] Thomas EM , Popple RA , Wu X , et al. Comparison of plan quality and delivery time between volumetric arc therapy (RapidArc) and Gamma Knife radiosurgery for multiple cranial metastases. Neurosurgery. 2014;75(4):409‐17. doi:10.1227/NEU.0000000000000448 24871143 10.1227/NEU.0000000000000448 PMC4203364

