# Supplementary material for: Modeling dose to normal brain following hypofractionated stereotactic radiotherapy to a single brain metastasis
Source: J Appl Clin Med Phys. 2026 Feb 23;27(3):e70468. doi: 10.1002/acm2.70468 (PMC12928994; doi:10.1002/acm2.70468)
Supplement: Supplementary file 1 — Supporting Information [file ACM2-27-e70468-s001.docx]

**SUPPLEMENTAL MATERIAL**

**Supplementary Data 1**: Initial patient-specific, dosimetric and treatment technique variables.

Patient Specific Variables

| Variable/Outcome | Description |
| --- | --- |
| Dose | Reference dose |
| Fractions | Number of treatment fractions |
| SIB | Was there a simultaneous integrated boost? (SIB) |
| Coverage | What percentage of the reference dose is covering the PTV? |
| PTV_Volume | What is the volume of the PTV in cm³? |
| Normal_Brain_Volume | What is the volume of normal brain (Brain-PTV) in cm³? |
| Brain_Volume | What is the total volume of brain in cm³? |
| Brain_Avoid_Volume | What is the total brain avoid (Brain-[PTV+1cm]) in cm³? |
| PTV_Location | Is the PTV superficial or deep? |
| Number of Organ at Risks (OARs) ? | What is the total number of OARs that have a goal requested by the RO? |
| Number of PRVs? | What is the total number of PRVs that have a goal requested by the RO? |
| Number of OARs and PRVs? | What is the total number of OARs and PRVs that have a goal requested by the RO? |
| Any competing OARs? | Is there a competing OAR? (Does the OAR abut the PTV with the same tolerance as the reference dose) |
| Brain Avoid Max dose constraint? | What is the Brain Avoid max dose constraint requested by the RO? |
| Brain Avoid Mean dose constraint? | What is the Brain Avoid mean dose constraint requested by the RO? |
| PTV_shape | What is the shape of the PTV structure? Spherical or irregular? |

**Treatment Technique Variables**

| Variable/Outcome | Description |
| --- | --- |
| Arc_Numbers | Total number of arcs? |
| Protocol | Which protocol is the planner using? (Old, new or other) |
| Coplanar? | Is the plan coplanar? Y or N |
| Arc_Angle_Start_1 | What angle did the first arc start at? |
| Arc_Angle_Stop_1 | What angle did the first arc finish at? |
| Arc_Angle_Start_2 | What angle did the second arc start at? |
| Arc_Angle_Stop_2 | What angle did the second arc finish at? |
| Arc_Angle_Start_3 | What angle did the third arc start at? |
| Arc_Angle_Stop_3 | What angle did the third arc finish at? |
| Arc_Angle_Start_4 | What angle did the fourth arc start at? |
| Arc_Angle_Stop_4 | What angle did the fourth arc finish at? |
| Collimator_Arc_1 | What angle is the collimator on for arc 1? |
| Collimator_Arc_2 | What angle is the collimator on for arc 2? |
| Collimator_Arc_3 | What angle is the collimator on for arc 3? |
| Collimator_Arc_4 | What angle is the collimator on for arc 4? |
| Number of Collimator Angles | How many different collimator angles does the plan have? |
| Floor_Angle_Arc_1 | What is the floor angle for arc 1? |
| Floor_Angle_Arc_2 | What is the floor angle for arc 2? |
| Floor_Angle_Arc_3 | What is the floor angle for arc 3? |
| Floor_Angle_Arc_4 | What is the floor angle for arc 4? |
| Number of Floor Angles? | How many different floor angles does the plan have? |
| Arc_Direction_1 | Which direction is arc 1 going? ACW or CW? |
| Arc_Direction_2 | Which direction is arc 2 going? ACW or CW? |
| Arc_Direction_3 | Which direction is arc 3 going? ACW or CW? |
| Arc_Direction_4 | Which direction is arc 4 going? ACW or CW? |
| Arc_Length_1 | How long (in degrees) is arc number 1? |
| Arc_Length_2 | How long (in degrees) is arc number 2? |
| Arc_Length_3 | How long (in degrees) is arc number 3? |
| Arc_Length_4 | How long (in degrees) is arc number 4? |
| Arc_Length_Total | What is the cumulative total arc length? (in degrees) |

**Plan Metric Variables**

| Variable/Outcome | Description |
| --- | --- |
| dMax | What is the absolute point max dose in Gy? |
| dMax_% | What is the absolute point max dose as a percentage of the reference dose? |
| MU/# (Arc 1) | What is the total monitor units of arc 1? |
| MU/# (Arc 2) | What is the total monitor units of arc 2? |
| MU/# (Arc 3) | What is the total monitor units of arc 3? |
| MU/# (Arc 4) | What is the total monitor units of arc 4? |
| MU (total) | What is the cumulative MU total of all arcs? |
| Delivery Time (Arc 1) | What is the delivery time of arc 1? |
| Delivery Time (Arc 2) | What is the delivery time of arc 2? |
| Delivery Time (Arc 3) | What is the delivery time of arc 3? |
| Delivery Time (Arc 4) | What is the delivery time of arc 4? |
| BrainAvoidMaxGy | What was the resulting Brain Avoid max dose in Gy accepted by the RO? |
| BrainAvoidMax%PD | What was the resulting Brain Avoid max dose (as a % of the reference dose) accepted by the RO? |
| MeanNormalBrain | What was the resulting normal brain mean dose accepted by the RO? |
| IntegralNormalBrain | What was the integral normal brain? (in Gy/cm³) |
| MeanBrainAvoid | What was the resulting brain avoid mean dose accepted by the RO? |
| IntegralBrainAvoid | What was the integral brain avoid? (in Gy/cm³) |
| V50%ofPD | What volume of normal brain received 50% of the prescribed dose? (in cm³) |
| V25%ofPD | What volume of normal brain received 25% of the prescribed dose? (in cm³) |
| V10%ofPD | What volume of normal brain received 10% of the prescribed dose? (in cm³) |

**Supplementary Data 2:** Bivariate testing outcomes for three model outcomes

**Normal Brain Mean Dose in Gy**

| **Outcome** | **Variables** | **Test** | **P value** |
| --- | --- | --- | --- |
| Mean Brain Dose | PTV Location | Independent T-Test | p<0.001 |
| Mean Brain Dose | Total Number of Arcs | Independent T-Test | p=0.001 |
| Mean Brain Dose | Total number of Rings | Independent T-Test | p=0.924 |
| Mean Brain Dose | Tumour Shape | Mann-Whitney Test | p<0.001 |
| Mean Brain Dose | Is there a competing OAR? | Mann-Whitney Test | p=0.046 |
| Mean Brain Dose | Total number of different collimator angles | Independent T-Test | p=0.08 |
| Mean Brain Dose | Total number of different floor angles | Kruskal-Wallis Test | p=0.07 |
| Mean Brain Dose | Total Arc Length | Pearson’s Correlation | R=0.39, p<0.001 |
| Mean Brain Dose | Total MU | Pearson’s Correlation | R= -0.14, p=0.17 |
| Mean Brain Dose | Total OARs | Pearson’s Correlation | R=0.08, p=0.44 |
| Mean Brain Dose | PTV volume | Pearson’s Correlation | R=0.78, p<0.001 |
| Mean Brain Dose | Arc Combinations | ANOVA | p=0.127 |

**Volume of normal brain receiving 50% of the prescribed dose**

| **Outcome** | **Variables** | **Test** | **P value** |
| --- | --- | --- | --- |
| V50% | PTV Location | Independent T-Test | p=0.003 |
| V50% | Total Number of Arcs | Independent T-Test | p=0.002 |
| V50% | Total Number of Rings | Independent T-Test | p=0.76 |
| V50% | Tumour Shape | Mann Whitney U Test | p<0.001 |
| V50% | Is there a competing OAR? | Mann Whitney U Test | p=0.026 |
| V50% | Total Number of different collimator angles | Independent T-Test | p=0.008 |
| V50% | Total number of different floor angles | Kruskal Wallis | p=0.50 |
| V50% | Total Arc Length | Pearson’s Correlation | R=0.44, p<0.001 |
| V50% | Total MU | Pearson’s Correlation | R= -0.11, p=0.30 |
| V50% | Total OARs | Pearson’s Correlation | R=0.04, p=0.73 |
| V50% | PTV volume | Pearson’s Correlation | R=0.84, p<0.001 |
| V50% | Arc Combinations | ANOVA | p=0.416 |

**Volume of normal brain receiving 25% of the prescribed dose**

| **Outcome** | **Variables** | **Test** | **P value** |
| --- | --- | --- | --- |
| V25% | PTV Location | Independent T-Test | p=0.001 |
| V25% | Total Arc Number | Independent T-Test | p=0.004 |
| V25% | Total Number of Rings | Independent T-Test | p=0.368 |
| V25% | Tumour Shape | Mann Whitney | p<0.001 |
| V25% | Is there a competing OAR | Mann Whitney | p=0.021 |
| V25% | Total number of collimator angles | Independent T Test | p=0.015 |
| V25% | Total number of different floor angles | Kruskal-Wallis Test | p=0.161 |
| V25% | Total arc length | Pearson’s Correlation | R=0.45, p<0.001 |
| V25% | Total MU | Pearson’s Correlation | R= -0.08, p=0.480 |
| V25% | Total OAR | Pearson’s Correlation | R=0.10, p=0.335 |
| V25% | PTV volume | Pearson’s Correlation | R=0.81, p<0.001 |
| V25% | Arc Combinations | ANOVA | p=0.667 |

**Supplementary Data 3: MLR testing outcomes for the three model outcomes**

**Normal Brain Mean**

This section will cover the steps taken in the MLR statistical test for the model outcome of normal brain mean in Gy. The model started with all nine variables listed above.

Table 31: MLR Run 1 for Normal Brain Mean Dose in Gy

| Variable | Significance |
| --- | --- |
| PTV Location | <0.001 |
| **Floor Angles** | **0.998** |
| Arc Combinations | 0.696 |
| PTV Volume | <0.001 |
| PTV Shape | 0.011 |
| Arc Number | 0.292 |
| Competing OAR | 0.984 |
| Collimator Angles | 0.722 |
| Arc Length Total | 0.868 |

Total number of different floor angles was removed as it had the largest p value of 0.998 and the model was rerun with the remaining 8 variables. Adjusted R² was 66.8%.

Table 32: MLR Run 2 for Normal Brain Mean Dose in Gy

| Variable | Significance |
| --- | --- |
| PTV Location | <0.001 |
| Collimator Angles | 0.722 |
| Arc Combinations | 0.928 |
| PTV Volume | <0.001 |
| PTV Shape | 0.011 |
| Arc Number | 0.292 |
| **Competing OAR** | **0.984** |
| Arc Length Total | 0.868 |

Competing OAR was removed as it had the largest p value of 0.984 and the model was rerun with the remaining 7. Adjusted R² was 66.8%.

Table 33: MLR Run 3 for Normal Brain Mean Dose in Gy

| Variable | Significance |
| --- | --- |
| PTV Location | <0.001 |
| Collimator Angles | 0.713 |
| **Arc Combinations** | **0.925** |
| PTV Volume | <0.001 |
| PTV Shape | 0.010 |
| Arc Number | 0.286 |
| Arc Length Total | 0.858 |

Arc Combinations was removed as it had the largest p value of 0.925 and the model was rerun with the remaining 6 variables. Adjusted R² was 67.2%.

Table 34: MLR Run 4 for Normal Brain Mean in Gy

| Variable | Significance |
| --- | --- |
| PTV Location | <0.001 |
| Collimator Angles | 0.627 |
| PTV Volume | <0.001 |
| PTV Shape | 0.005 |
| Arc Number | 0.136 |
| **Total Arc Length** | **0.855** |

Total Arc Length was removed as it had the largest p value of 0.855 and the model was rerun with the remaining 5 variables. Adjusted R² was 68.4%.

Table 35: MLR Run 5 for Normal Brain Mean in Gy

| Variable | Significance |
| --- | --- |
| PTV Location | <0.001 |
| **Collimator Angles** | **0.619** |
| PTV Volume | <0.001 |
| PTV Shape | 0.004 |
| Arc Number | 0.113 |

Collimator Angles was removed as it had the largest p value of 0.619 and the model was rerun with the remaining 4 variables. Adjusted R² was 68.8%.

Table 36: MLR Run 6 for Normal Brain Mean in Gy

| Variable | Significance |
| --- | --- |
| PTV Location | <0.001 |
| PTV Volume | <0.001 |
| Tumour Shape | 0.004 |
| **Arc Number** | **0.128** |

Arc Number was removed as it had the largest p value of 0.128 and the model was rerun with the remaining 3 variables. Adjusted R² was 69.1%.

Table 37: MLR Run 7 for Normal Brain Mean in Gy

| Variable | Significance |
| --- | --- |
| PTV Location | <0.001 |
| PTV Volume | <0.000 |
| PTV Shape | 0.004 |

After completing run 7, all variables were significant. Adjusted R² was 68.6%. The MLR was then performed including those three variables. The parameter estimates which provide the coefficients for the model and the referent groups is provided in Table 38 below:

Table 38: Parameter Estimates for MLR Normal Brain Mean Dose in Gy with final variables selected.

| Parameter | B | Significance | 95% Confidence Interval Lower Bound | 95% Confidence Interval Upper Bound |
| --- | --- | --- | --- | --- |
| Intercept | 1.70 | <0.001 | 1.49 | 1.91 |
| PTV Location 1 | 0.55 | <0.001 | 0.30 | 0.80 |
| PTV Location 2 | 0 (Referent) |  |  |  |
| PTV Shape 1 | -0.47 | 0.004 | -0.78 | -0.16 |
| PTV Shape 2 | 0 (Referent) |  |  |  |
| PTV Volume | 0.02 | <0.001 | 0.02 | 0.03 |

**V50%**

This section will cover the steps taken in the MLR for the model outcome of volume of normal brain receiving 50% of the prescribed dose. The model started with all nine variables selected from bivariate testing.

Table 39: MLR Run 1 for V50%

| Variable | Significance |
| --- | --- |
| PTV Location | <0.001 |
| PTV Shape | 0.002 |
| Arc Combination | 0.001 |
| Competing OAR | 0.696 |
| Arc Number | 0.361 |
| Collimator Angles | 0.087 |
| **Floor Angles** | **0.801** |
| Arc Length Total | 0.564 |
| PTV Volume | <0.001 |

Total number of different floor angles was removed as it had the largest p value of 0.801 and the model was rerun with the remaining 8 variables. Adjusted R² was 78.4%.

Table 40: MLR Run 2 for V50%

| Variable | Significance |
| --- | --- |
| PTV Location | <0.001 |
| PTV Shape | 0.002 |
| Arc Combination | 0.005 |
| **Competing OAR** | **0.696** |
| Arc Number | 0.361 |
| Collimator Angles | 0.087 |
| Arc Length Total | 0.564 |
| PTV Volume | <0.001 |

Competing OAR was removed as it had the largest p value of 0.696 and the model was rerun with the remaining 7 variables. Adjusted R² was 78.3%.

Table 41: MLR Run 3 for V50%

| Variable | Significance |
| --- | --- |
| PTV Location | <0.001 |
| PTV Shape | 0.002 |
| Arc Combination | 0.005 |
| Arc Number | 0.331 |
| Collimator Angles | 0.056 |
| **Arc Length Total** | **0.475** |
| PTV Volume | <0.001 |

Arc Length Total was removed as it had the largest p value of 0.475 and the model was rerun with the remaining 6 variables. Adjusted R² was 78.6%.

Table 42: MLR Run 4 for V50%

| Variable | Significance |
| --- | --- |
| PTV Location | <0.001 |
| PTV Shape | 0.002 |
| Arc Combination | 0.002 |
| Collimator Angles | 0.059 |
| **Arc Number** | **0.405** |
| PTV Volume | <0.001 |

Arc Number was removed as it had the largest p value of 0.404 and the model was rerun with the remaining 5 variables. Adjusted R² was 78.7%.

Table 43: MLR Run 5 for V50%

| Variable | Significance |
| --- | --- |
| PTV Location | <0.001 |
| PTV Shape | 0.002 |
| Arc Combination | 0.002 |
| **Collimator Angles** | **0.088** |
| PTV Volume | <0.001 |

Total number of collimator angles was removed as it had the largest p value of 0.088 and the model was rerun with the remaining 4 variables. Adjusted R² was 78.8%.

Table 44: MLR Run 6 for V50%

| Variable | Significance |
| --- | --- |
| PTV Location | <0.001 |
| PTV Shape | 0.001 |
| Arc Combination | 0.003 |
| PTV Volume | <0.001 |

After completing run 6, all four variables kept were significant. Adjusted R² was 78.3%.

The MLR was then performed including those four variables. The parameter estimates which provide the coefficients for the model and the referent groups is provided in table 45 below.

Table 45: Parameter Estimates for MLR V50% with final variables selected.

| Parameter | B | Significance | 95% Confidence Interval Lower Bound | 95% Confidence Interval Upper Bound |
| --- | --- | --- | --- | --- |
| Intercept | 17.86 | <0.001 | 8.49 | 27.23 |
| PTV Location 1 | 10.48 | <0.001 | 5.76 | 15.21 |
| PTV Location 2 | 0 (Referent) |  |  |  |
| Arc Combination 1 | 7.45 | 0.180 | -3.52 | 18.42 |
| Arc Combination 2 | 0.20 | 0.964 | -8.68 | 9.09 |
| Arc Combination 3 | 11.94 | 0.022 | 1.75 | 22.13 |
| Arc Combination 4 | -0.82 | 0.863 | -10.29 | 8.65 |
| Arc Combination 5 | 0 (Referent) |  |  |  |
| PTV Shape 1 | -9.70 | 0.001 | -15.51 | -3.89 |
| PTV Shape 2 | 0 (Referent) |  |  |  |
| PTV Volume | 0.61 | <0.001 | 0.52 | 0.70 |

**V25%**

This sub-section will cover the steps taken in the MLR for the model outcome of volume of normal brain receiving 25% of the prescribed dose. The model started with all nine variables selected from bivariate testing.

Table 46: MLR Run 1 for V25%

| Variable | Significance |
| --- | --- |
| PTV Location | <0.001 |
| PTV Shape | 0.020 |
| Arc Combination | 0.053 |
| Competing OAR | 0.863 |
| Arc Number | 0.799 |
| Collimator Angles | 0.205 |
| **Floor Angles** | **0.886** |
| Arc Length Total | 0.471 |
| PTV Volume | <0.001 |

Total number of different floor angles was removed as it had the largest p value of 0.886 and the model was rerun with the remaining 8 variables. Adjusted R² was 72.7%.

Table 47: MLR Run 2 for V25%

| Variable | Significance |
| --- | --- |
| PTV Location | <0.001 |
| PTV Shape | 0.020 |
| Arc Combination | 0.064 |
| **Competing OAR** | **0.863** |
| Arc Number | 0.799 |
| Collimator Angles | 0.205 |
| Arc Length Total | 0.471 |
| PTV Volume | <0.001 |

Competing OAR was removed as it had the largest p value of 0.863 and the model was rerun with the remaining 7 variables. Adjusted R² was 72.7%.

Table 48: MLR Run 3 for V25%

| Variable | Significance |
| --- | --- |
| PTV Location | <0.001 |
| PTV Shape | 0.019 |
| Arc Combination | 0.062 |
| **Arc Number** | **0.780** |
| Collimator Angles | 0.167 |
| Arc Length Total | 0.422 |
| PTV Volume | <0.001 |

Total number of arcs was removed as it had the largest p value of 0.780 and the model was rerun with the remaining 6 variables. Adjusted R² was 73%.

Table 49: MLR Run 4 for V25%

| Variable | Significance |
| --- | --- |
| PTV Location | <0.001 |
| PTV Shape | 0.019 |
| Arc Combination | 0.056 |
| Collimator Angles | 0.159 |
| **Total Arc Length** | **0.445** |
| PTV Volume | <0.001 |

Total Arc Length was removed as it had the largest p value of 0.445 and the model was rerun with the remaining 5 variables. Adjusted R² was 73.4%.

Table 50: MLR Run 5 for V25%

| Variable | Significance |
| --- | --- |
| PTV Location | <0.001 |
| PTV Shape | 0.024 |
| Arc Combination | 0.028 |
| **Collimator Angles** | **0.143** |
| PTV Volume | <0.001 |

Total number of collimator angles was removed as it had the largest p value of 0.143 and the model was rerun with the remaining 4 variables. Adjusted R² was 73.5%.

Table 51: MLR Run 6 for V25%

| Variable | Significance |
| --- | --- |
| PTV Location | <0.001 |
| PTV Shape | 0.019 |
| Arc Combination | 0.035 |
| PTV Volume | <0.001 |

After completing run 6, all four variables kept were significant. Adjusted R² was 73.1%.

The MLR was then performed including those four variables. The parameter estimates which provide the coefficients for the model and the referent groups is provided in table 52 below.

Table 52: Parameter Estimates for MLR V25% with final variables selected

| Parameter | B | Significance | 95% Confidence Interval Lower Bound | 95% Confidence Interval Upper Bound |
| --- | --- | --- | --- | --- |
| Intercept | 53.5 | 0.002 | 8.49 | 27.23 |
| PTV Location 1 | 39.61 | <0.001 | 5.76 | 15.21 |
| PTV Location 2 | 0 (Referent) |  |  |  |
| Arc Combination 1 | 30.31 | 0.123 | -3.52 | 18.42 |
| Arc Combination 2 | 3.02 | 0.848 | -8.68 | 9.09 |
| Arc Combination 3 | 28.06 | 0.125 | 1.75 | 22.13 |
| Arc Combination 4 | -5.25 | 0.755 | -10.29 | 8.65 |
| Arc Combination 5 | 0 (Referent) |  |  |  |
| PTV Shape 1 | -24.71 | 0.019 | -15.51 | -3.89 |
| PTV Shape 2 | 0 (Referent) |  |  |  |
| PTV Volume | 1.87 | <0.001 | 0.52 | 0.70 |
